# Supplementary material for: Structural Insights into Kainate Receptor Desensitization
Source: bioRxiv. 2025 Apr 23:2025.03.27.645769. Originally published 2025 Mar 31. Preprint. [Version 2] doi: 10.1101/2025.03.27.645769 (PMC11996427; doi:10.1101/2025.03.27.645769)
Supplement: Supplement 1 — Figure S1. Design of the double cysteine crosslink mutant of GluK2. (A) Schematic illustration of the KAR subunit topology. Each KAR subunit is composed of four domains: ATD, LBD,TMD, and CTD. The LBD contains two segments termed D1 (upper lobe) and D2 (lower lobe). Agonists bind to the interface between the D1 and D2 lobes of the LBD. (B) Homology model of the active GluK2 KAR, generated based on the active GluA2 AMPAR (PDB code 5WEO) as a template. (C) The inter-protomer interface between subunit A and subunit B in the homology model. (D) The distance between residues K676 and N802 in the BPAM344-, concanavalin A-, and glutamate-bound open/active GluK2 (PDB code 9B36)27, BPAM344-bound (without orthosteric ligands) inactive GluK2 (PDB code 8FWS)32, and glutamate-bound deep desensitized GluK2 (PDB code 9B38)27 structures. (E, F) Two designed disulfide crosslinks at the inter-protomer interface, shown in the homology model and the schematic figure. Figure S2. Channel properties of the single cysteine mutant and protein preparation. (A) Representative electrophysiological traces from HEK293T cells expressing GluK2 WT, GluK2 K676C, and GluK2 N802C, activated by 10 mM glutamate. (B) Size exclusion chromatography profile of the GluK2 N802C/K676C mutant eluted from the Superose 6 column chromatography. The fractions used for the cryo-EM studies are indicated by the black dotted line. (C) SDS-PAGE analysis of the purified GluK2 N802C/K676C mutant, with the black line indicating the full-length GluK2 protein band. Figure S3. Cryo-EM data processing workflow for structures of the GluK2 K676C/N802C mutant in the presence of glutamate and BPAM344 (a), and glutamate alone (b) Figure S4. Cryo-EM analysis of GluK2 K676C/N802C. (A, D) Local resolution maps, (B, E) Fourier shell correlation (FSC) curves. The threshold was set at 0.143 for unmasked and masked curves, and a 0.5 threshold was used for the map-to-model curve. (C, F) Angular distribution of particles calculated using th [file media-1.pdf]

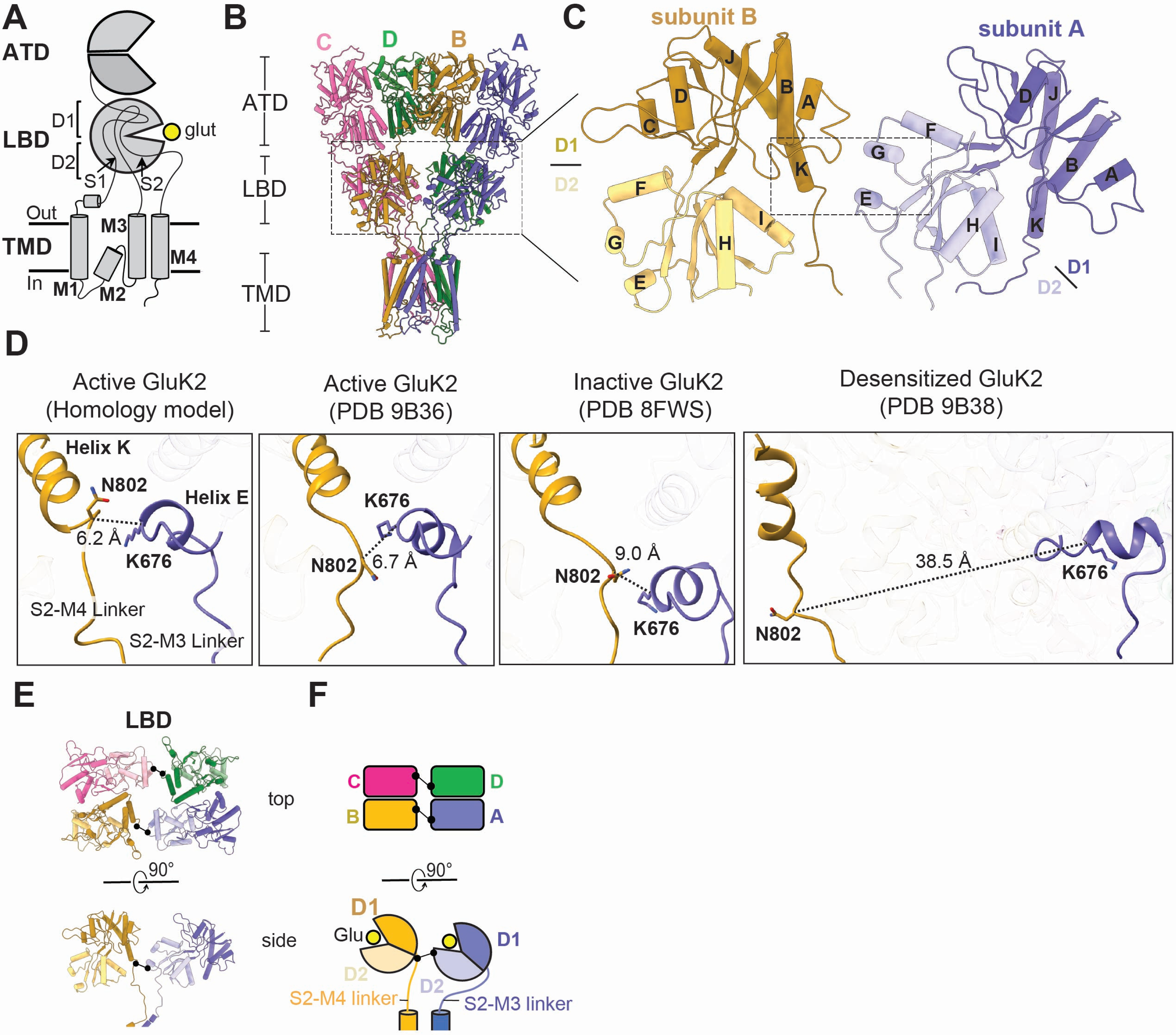
A
ATD
D1
LBD
glut
D2
S1
S2
Out
M3
TMD
M4
In
M1
M2
B
C
D
B
A
ATD
LBD
TMD
C
subunit B
subunit A
D1
D2
D1
D2
D
Active GluK2
(Homology model)
Active GluK2
(PDB 9B36)
Inactive GluK2
(PDB 8FWS)
Desensitized GluK2
(PDB 9B38)
Helix K
N802
Helix E
6.2 Å
K676
S2-M4 Linker
S2-M3 Linker
K676
N802
6.7 Å
9.0 Å
N802
K676
38.5 Å
K676
N802
E
LBD
top
90°
side
F
C
D
B
A
90°
D1
Glu
D1
D2
D2
S2-M4 linker
S2-M3 linker

Figure S2

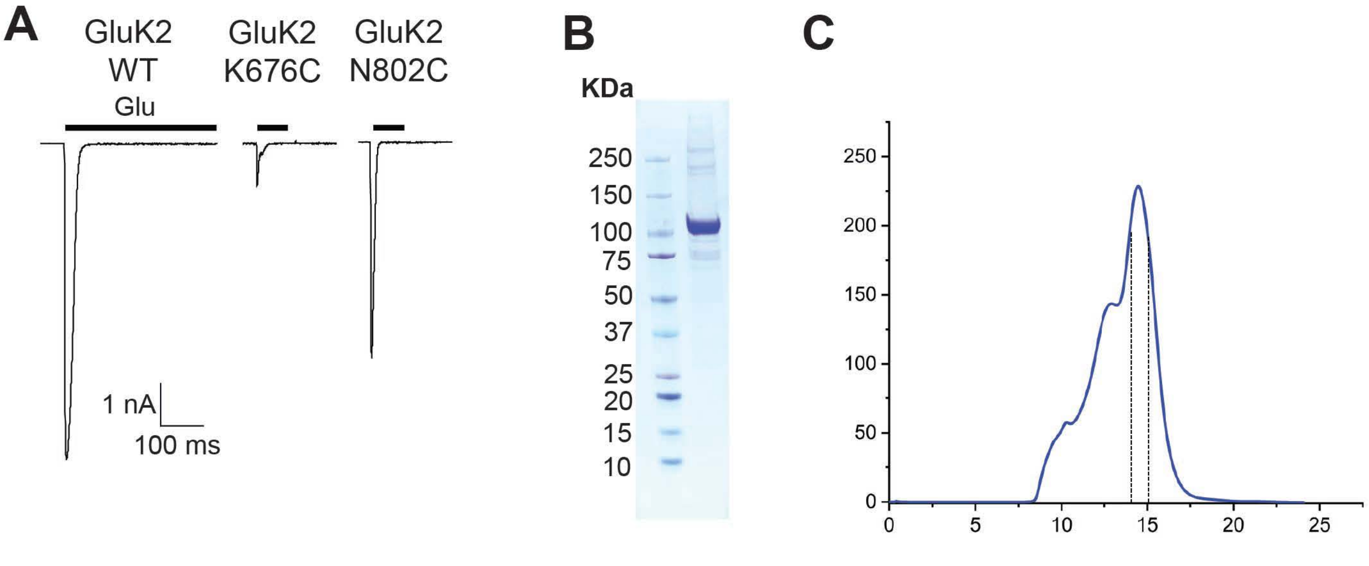
A
GluK2 WT
GluK2 K676C
GluK2 N802C
Glu
1 nA
100 ms
B
KDa
250
150
100
75
50
37
25
20
15
10
C
250
200
150
100
50
0
0
5
10
15
20
25

Figure S2

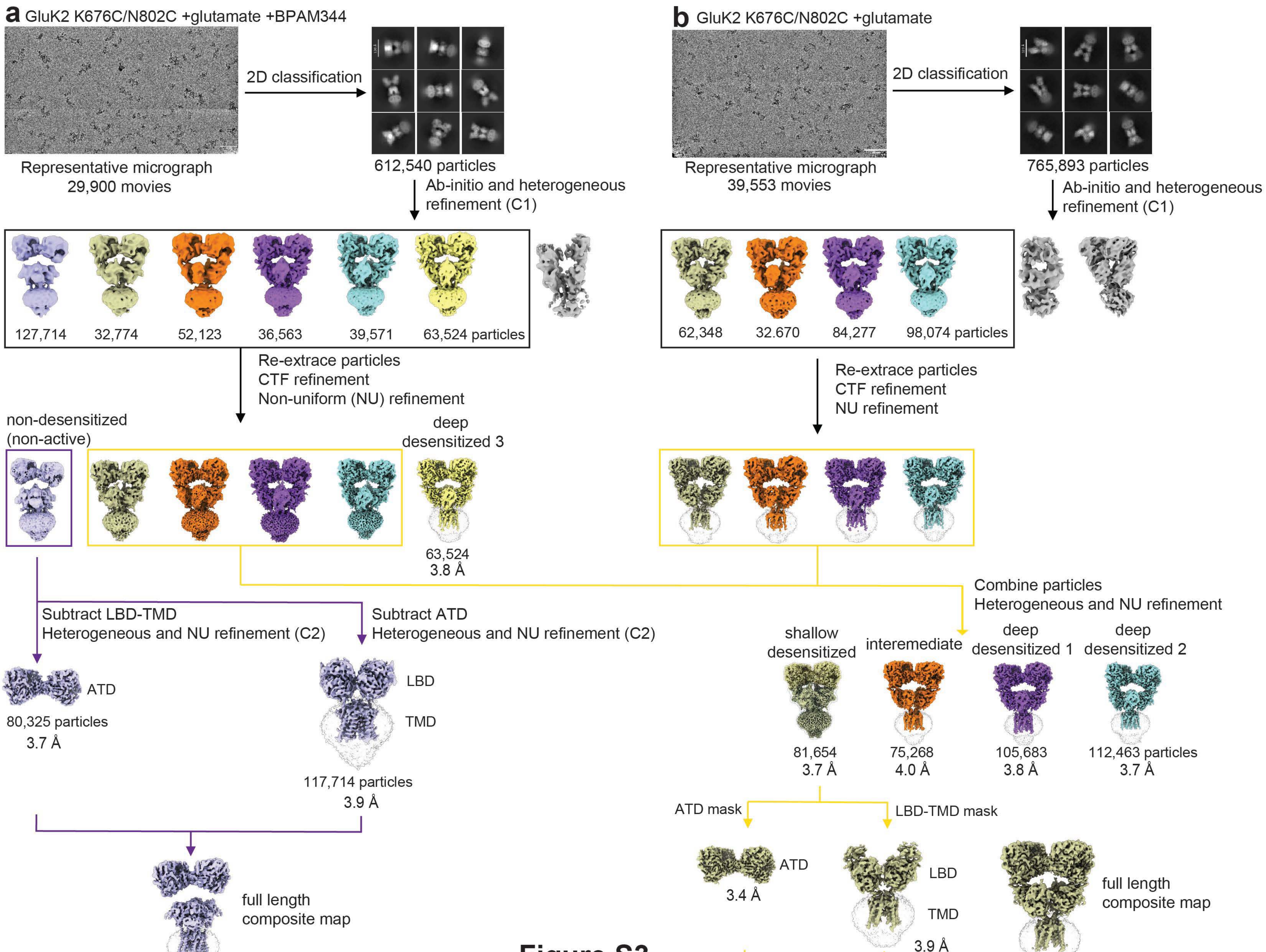
a GluK2 K676C/N802C +glutamate +BPAM344
2D classification
Representative micrograph
29,900 movies
612,540 particles
Ab-initio and heterogeneous refinement (C1)
127,714
32,774
52,123
36,563
39,571
63,524 particles
Re-extrace particles
CTF refinement
Non-uniform (NU) refinement
non-desensitized (non-active)
deep desensitized 3
63,524
3.8 Å
Subtract LBD-TMD
Heterogeneous and NU refinement (C2)
Subtract ATD
Heterogeneous and NU refinement (C2)
ATD
80,325 particles
3.7 Å
LBD
TMD
117,714 particles
3.9 Å
full length composite map
b GluK2 K676C/N802C +glutamate
2D classification
Representative micrograph
39,553 movies
765,893 particles
Ab-initio and heterogeneous refinement (C1)
62,348
32.670
84,277
98,074 particles
Re-extrace particles
CTF refinement
NU refinement
Combine particles
Heterogeneous and NU refinement
shallow desensitized
interemediate
deep desensitized 1
deep desensitized 2
81,654
3.7 Å
75,268
4.0 Å
105,683
3.8 Å
112,463 particles
3.7 Å
ATD mask
LBD-TMD mask
ATD
3.4 Å
LBD
TMD
3.9 Å
full length composite map

Figure S3

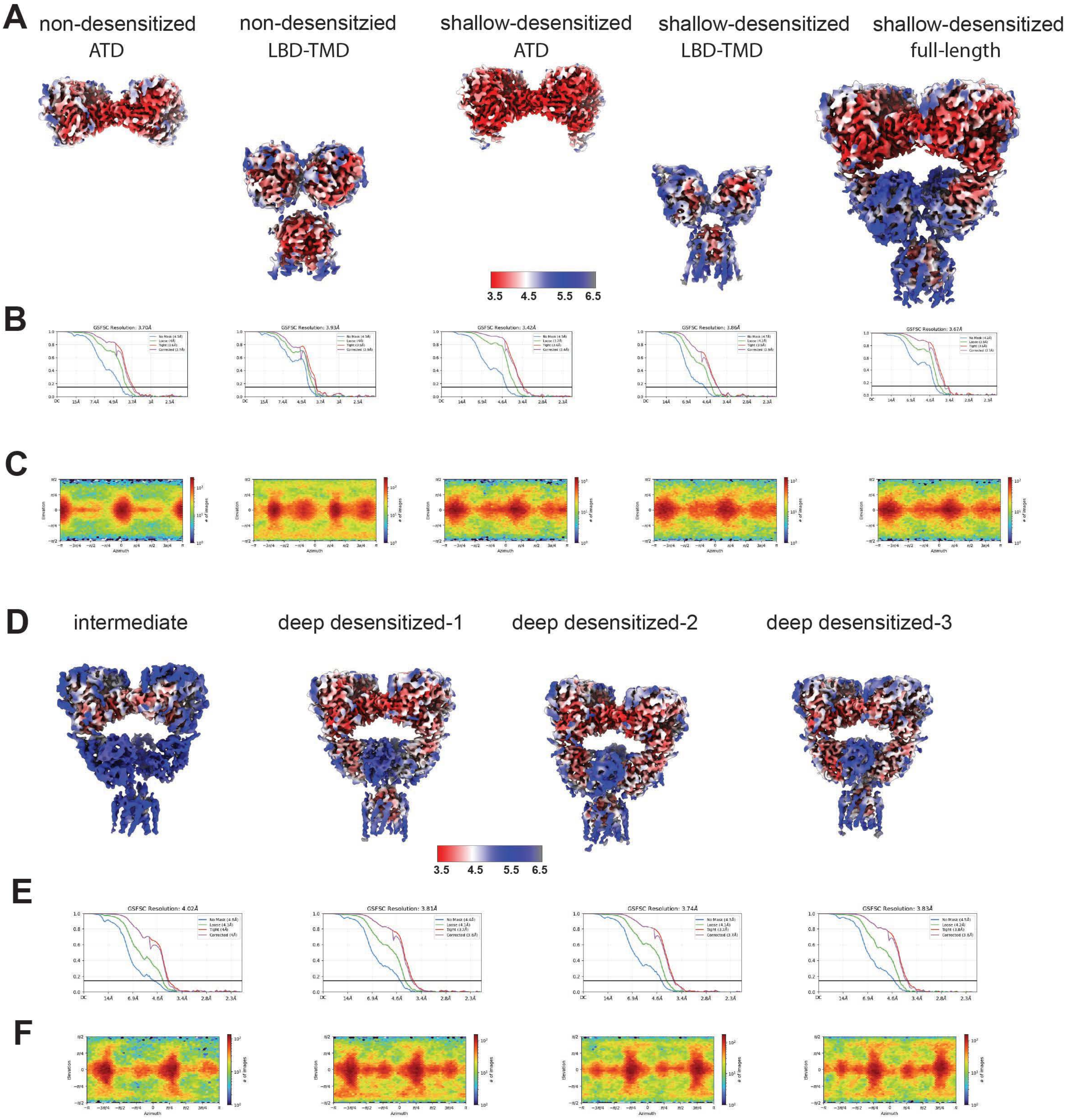
A
non-desensitized ATD
non-desensitzied LBD-TMD
shallow-desensitized ATD
shallow-desensitized LBD-TMD
shallow-desensitized full-length
3.5 4.5 5.5 6.5
B
GSFSC Resolution: 3.70Å
GSFSC Resolution: 3.93Å
GSFSC Resolution: 3.42Å
GSFSC Resolution: 3.86Å
GSFSC Resolution: 3.67Å
C
D
intermediate
deep desensitized-1
deep desensitized-2
deep desensitized-3
3.5 4.5 5.5 6.5
E
GSFSC Resolution: 4.02Å
GSFSC Resolution: 3.81Å
GSFSC Resolution: 3.74Å
GSFSC Resolution: 3.83Å
F

Figure S4

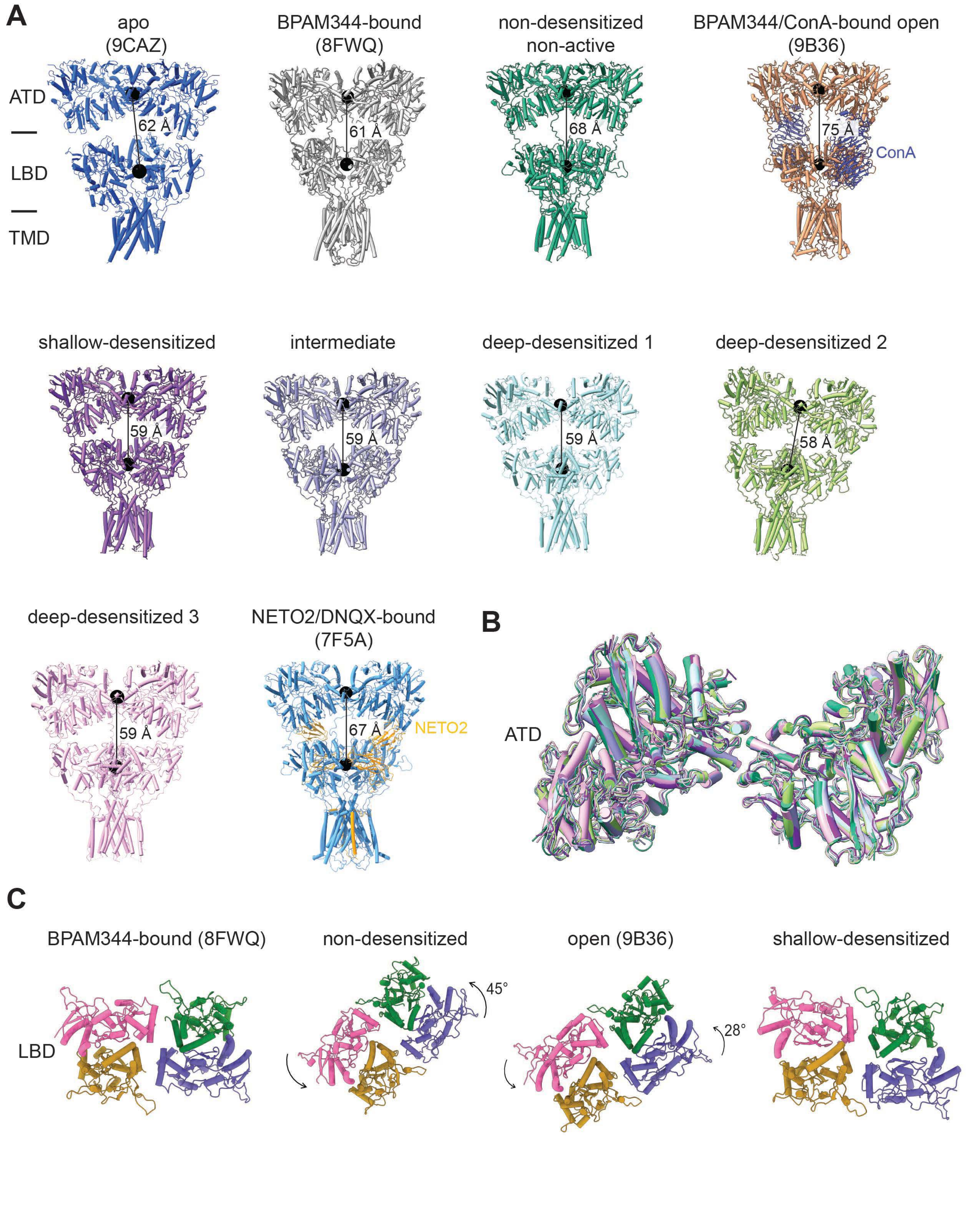

A
apo (9CAZ)
BPAM344-bound (8FWQ)
non-desensitized non-active
BPAM344/ConA-bound open (9B36)
ATD
LBD
TMD
62 Å
61 Å
68 Å
75 Å
ConA
shallow-desensitized
intermediate
deep-desensitized 1
deep-desensitized 2
59 Å
59 Å
59 Å
58 Å
deep-desensitized 3
NETO2/DNQX-bound (7F5A)
59 Å
67 Å
NETO2
B
ATD
C
BPAM344-bound (8FWQ)
non-desensitized
open (9B36)
shallow-desensitized
LBD
45°
28°

Figure S5

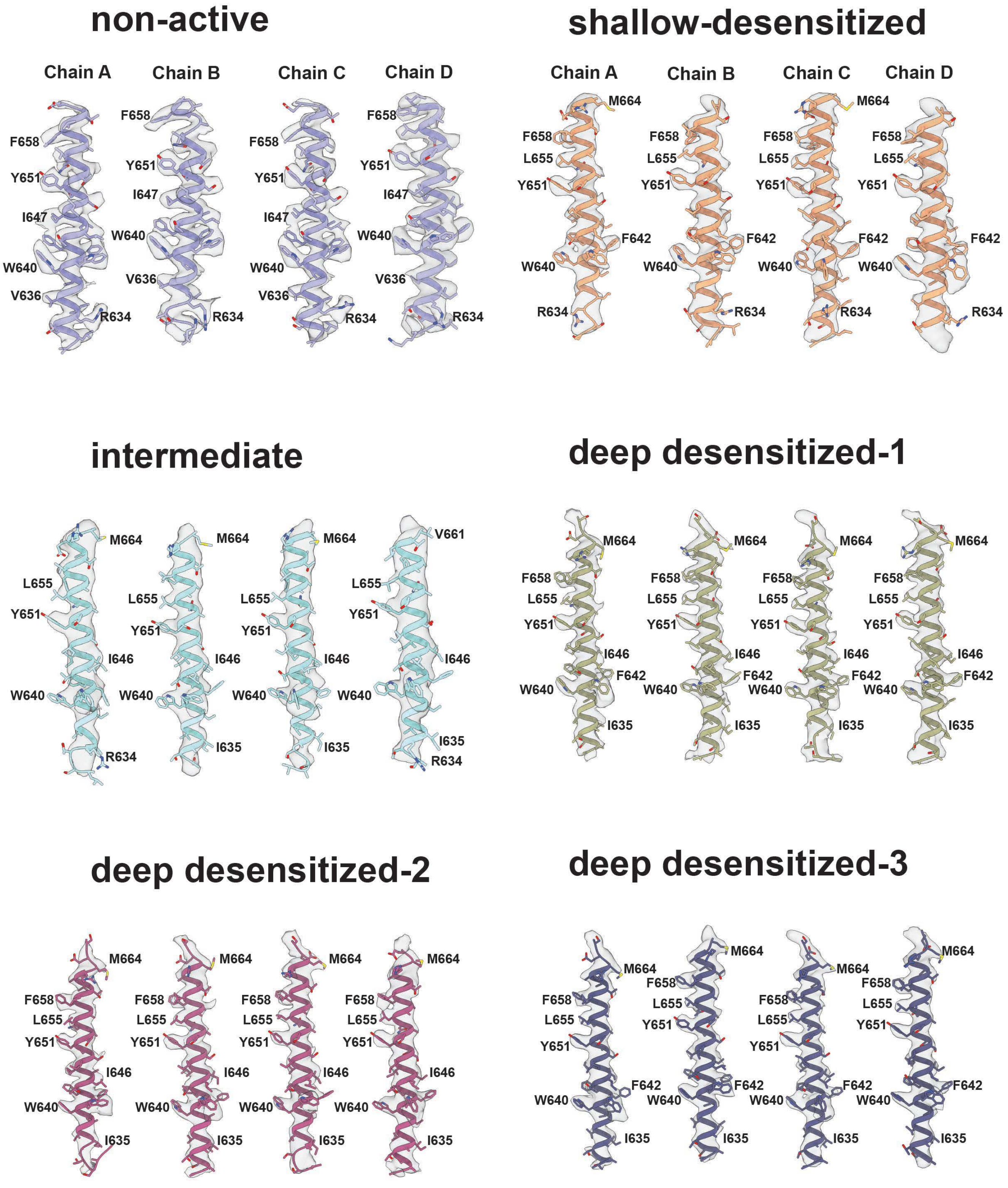
non-active
Chain A
Chain B
Chain C
Chain D
F658
Y651
I647
W640
V636
R634
F658
Y651
I647
W640
V636
R634
F658
Y651
I647
W640
V636
R634
F658
Y651
I647
W640
V636
R634
shallow-desensitized
Chain A
Chain B
Chain C
Chain D
M664
F658
L655
Y651
F642
W640
R634
F658
L655
Y651
F642
W640
R634
M664
F658
L655
Y651
F642
W640
R634
F658
L655
Y651
F642
W640
R634
intermediate
M664
L655
Y651
I646
W640
R634
M664
L655
Y651
I646
W640
I635
M664
L655
Y651
I646
W640
I635
V661
L655
Y651
I646
W640
I635
R634
deep desensitized-1
M664
F658
L655
Y651
I646
F642
W640
I635
M664
F658
L655
Y651
I646
F642
W640
I635
M664
F658
L655
Y651
I646
F642
W640
I635
M664
F658
L655
Y651
I646
F642
W640
I635
deep desensitized-2
M664
F658
L655
Y651
I646
W640
I635
M664
F658
L655
Y651
I646
W640
I635
M664
F658
L655
Y651
I646
W640
I635
M664
F658
L655
Y651
I646
W640
I635
deep desensitized-3
M664
F658
L655
Y651
F642
W640
I635
M664
F658
L655
Y651
F642
W640
I635
M664
F658
L655
Y651
F642
W640
I635
M664
F658
L655
Y651
F642
W640
I635

Figure S6

Table S1. Cryo-EM data collection, refinement, and validation statistics.

	Non-desensitized ATD (EMD-48765) 9MZQ	Non-desensitized LBD-TMD (EMD-48766) 9MZR	Non-desensitized composite full-length (EMD-48767) 9MZS	Shallow-desensitized ATD (EMD-48762) 9MZN	Shallow-desensitized LBD-TMD (EMD-48763) 9MZO	Shallow-desensitized consensus full-length (EMD-48761) 9MZM	Shallow-desensitized composite full-length (EMD-48764) 9MZP
**Data collection and professing**							
Microscope	Titan Krios	Titan Krios	Titan Krios	Titan Krios	Titan Krios	Titan Krios	Titan Krios
Magnification (kx)	81	81	81	81	81	81	81
Voltage (kV)	300	300	300	300	300	300	300
Energy filter	Gatan	Gatan	Gatan	Gatan	Gatan	Gatan	Gatan
Energy filter slit width (eV)	20	20	20	20	20	20	20
Collection software	EPU	EPU	EPU	EPU	EPU	EPU	EPU
Electron exposure (e–/Å2)	50	50	50	50	50	50	50
Exposure rate (e–/Å2/frame)	1	1	1	1	1	1	1
Defocus range (μm)	-0.8 ~ -1.6	-0.8 ~ -1.6	-0.8 ~ -1.6	-0.8 ~ -1.6	-0.8 ~ -1.6	-0.8 ~ -1.6	-0.8 ~ -1.6
Pixel size (Å)	1.06	1.06	1.06	1.06	1.06	1.06	1.06
Symmetry imposed	C2	C2		C2	C2	C2	
Initial particle images (no.)	127,714	127,714	127,714	95,122	95,122	95.122	95,122
Final particle images (no.)	80,325	117,714	127,714	81.654	81,654	81.654	81.654
0.143 FSC half map masked (Å)	3.78	3.96		3.56	4.09	3.79	
0.143 FSC half map unmasked(Å)	3.97	4.05		3.71	4.19	3.96	
**Refinement**							
Refinement package	Phenix	Phenix	Phenix	Phenix	Phenix	Phenix	Phenix
Initial model used (PDB code)	9B36	9B36	9B36	9B36	9B36	9B36	9B36
0.5 FSC model resolution masked (Å)	3.87	4.19	4.19	3.7	4.23	4.14	3.83
0.5 FSC model resolution unmasked (Å)	4.13	4.28	4.29	3.84	4.32	4.22	4.03
Map sharpening B factor ($Å^2$)	-143	-163		-108	-141	-117.6	
Model composition							
Non-hydrogen atoms	12,582	11,927	25,004	12,784	11,857	24,825	24,825
Protein residues	1,534	1,501	3,093	1,565	1,489	3084	3,084
Ligand	23	4	29	24	9	30	30
B-factors (A^2)							
Protein	150.98	94.62	246.86	149.07	224.77	105.58	131.20
Ligand	173.91	35.82	240.10	144.34	188.59	103.13	135.65

R.m.s. deviations							
Bond lengths (Å)	0.004	0.003	0.002	0.003	0.004	0.003	0.002
Bond angles (°)	0.580	0.590	0.586	0.602	0.636	0.572	0.551
Validation							
MolProbity score	1.99	2.12	1.94	1.94	2.13	1.95	1.92
Clashscore	11.56	13.59	11.72	9.95	15.10	11.90	11.27
Rotamer outliers (%)	0	0.62	0.22	0.15	0.16	0.07	0
Ramachandran plot							
Favored (%)	93.84	92.30	94.83	93.64	93.08	94.85	94.88
Allowed (%)	6.16	7.70	5.17	6.36	6.92	5.15	5.12
Disallowed (%)	0	0	0	0	0	0	0
CaBLAM outliers (%)	3.49	3.07	2.52	2.65	3.50	3.18	3.41

	intermediate (EMD-48760) 9MZL	deep desensitized 1 (EMD-48759) 9MZK	deep desensitized 2 (EMD-48758) 9MZJ	deep desensitized 3 (EMD-48757) 9MZI
**Data collection and professing**				
Microscope	Titan Krios	Titan Krios	Titan Krios	Titan Krios
Magnification (kx)	81000X	81000X	81000X	81000X
Voltage (kV)	Gatan	Gatan	Gatan	Gatan
Energy filter	20	20	20	20
Energy filter slit width (eV)	EPU	EPU	EPU	EPU
Collection software	300	300	300	300
Electron exposure (e–/Å2)	50	50	50	50
Exposure rate (e–/Å2/frame)	1	1	1	1
Defocus range (μm)	-0.8 ~ -1.6	-0.8 ~ -1.6	-0.8 ~ -1.6	-0.8 ~ -1.6
Pixel size (Å)	1.06	1.06	1.06	1.06
Symmetry imposed	C1	C1	C1	C1
Initial particle images (no.)	96,3254	129,840	107,645	70,524
Final particle images (no.)	84793	120,840	97,645	63,524
0.143 FSC half map masked (Å)	4.16	3.94	3.89	4.26
0.143 FSC half map unmasked(Å)	4.31	4.16	4.09	4.20
**Refinement**				
Refinement package	Phenix	Phenix	Phenix	Phenix
Initial model used (PDB code)	9B39	9B39	9B39	9B39
0.5 FSC model resolution masked (Å)	4.28	4.13	4.06	4.14
0.5 FSC model resolution unmasked (Å)	4.40	4.28	4.24	4.29
Map sharpening B factor ($Å^2$)	-102	-104	-105	-107
Model composition				
Non-hydrogen atoms	23,785	24,372	24568	24,582
Protein residues	2,989	3,008	3,036	3,037
Ligand	11	45	44	44
B-factors (A^2)				
Protein	340.07	207.20	203.98	235.15
Ligand	239.79	199.40	233.78	228.36
R.m.s. deviations				
Bond lengths (Å)	0.003	0.003	0.003	0.003
Bond angles (°)	0.638	0.620	0.598	0.621

Validation				
MolProbity score	2.15	2.06	2.05	2.09
Clashscore	16.55	13.56	13.31	15.75
Rotamer outliers (%)	0.15	0	0.15	0.04
Ramachandran plot				
Favored (%)	93.43	93.71	93.61	94.24
Allowed (%)	6.54	6.29	6.36	5.76
Disallowed (%)	0.03	0	0.03	0
CaBLAM outliers (%)	4.07	3.44	3.26	3.13
